# Aberrant JAK/STAT Signaling Suppresses TFF1 and TFF2 through Epigenetic Silencing of *GATA6* in Gastric Cancer

**DOI:** 10.3390/ijms17091467

**Published:** 2016-09-02

**Authors:** Cheng-Shyong Wu, Kuo-Liang Wei, Jian-Liang Chou, Chung-Kuang Lu, Ching-Chuan Hsieh, Jora M. J. Lin, Yi-Fang Deng, Wan-Ting Hsu, Hui-Min David Wang, Chung-Hang Leung, Dik-Lung Ma, Chin Li, Michael W. Y. Chan

**Affiliations:** 1 Department of Gastroenterology and Hepatology, Chiayi Chang Gung Memorial Hospital, Chiayi 613, Taiwan; minjyun@cloud.cgmh.org.tw (C.-S.W.); wkliang@cgmh.org.tw (K.-L.W.); alien711021@gmail.com (J.-L.C.); cmusickimo@yahoo.com.tw (C.-K.L.); evonne8006@cgmh.org.tw (Y.-F.D.); 2 Department of Surgery, Chiayi Chang Gung Memorial Hospital, Chiayi 613, Taiwan; p12155@cgmh.org.tw; 3 Department of Life Science, National Chung Cheng University, 168 University Road, Min Hsiung, Chiayi 621, Taiwan; a584919@gmail.com (J.M.J.L.); white8020@hotmail.com (W.-T.H.); biocl@ccu.edu.tw (C.L.); 4 Department of Fragrance and Cosmetic Science, Kaohsiung Medical University, Kaohsiung 807, Taiwan; davidw@kmu.edu.tw; 5 State Key Laboratory of Quality Research in Chinese Medicine, Institute of Chinese Medical Sciences, University of Macau, Macau, China; duncanleung@umac.mo; 6 Department of Chemistry, Hong Kong Baptist University, Kowloon Tong, Hong Kong, China; edmondma@hkbu.edu.hk

**Keywords:** epigenetic silencing, gastric cancer, TFF1, TFF2, GATA6, STAT3

## Abstract

Aberrant Janus kinase (JAK)/signal transducer and activator of transcription (STAT) signaling is crucial to the development of gastric cancer. In this study, we examined the role of STAT3 in the expression and methylation of its targets in gastric cancer patients. Results from RNA sequencing identified an inverse correlation between the expression of *STAT3* and *GATA6* in 23 pairs of gastric cancer patient samples. We discovered that the expression of *GATA6* is epigenetically silenced through promoter methylation in gastric cancer cell lines. Interestingly, the inhibition of STAT3 using a novel STAT3 inhibitor restored the expression of *GATA6* and its targets, trefoil factors 1 and 2 (*TFF1/2*). Moreover, disruption of STAT3 binding to *GATA6* promoter by small hairpin RNA restored *GATA6* expression in AGS cells. A clinically significant correlation was also observed between the expression of *GATA6* and *TFF1/2* among tissue samples from 60 gastric cancer patients. Finally, bisulfite pyrosequencing revealed *GATA6* methylation in 65% (39/60) of the patients, and those with higher *GATA6* methylation tended to have shorter overall survival. In conclusion, we demonstrated that aberrant JAK/STAT signaling suppresses TFF1/2 partially through the epigenetic silencing of *GATA6*. Therapeutic intervention of STAT3 in reversing the epigenetic status of *GATA6* could benefit the treatment of gastric cancer and is worthy of further investigation.

## 1. Introduction

Gastric cancer is the fifth leading cause of cancer death in Taiwan, and infection with *Helicobacter pylori* is a major risk factor for gastric carcinoma [1]. Previous studies have shown that cytotoxin-associated gene A (CagA) positive *H. pylori* presents a greater risk of inducing gastric carcinogenesis than does CagA negative *H. pylori*. Studies have also shown that gastric epithelial cells infected with CagA positive *H. pylori* can activate the signal transducer and activator of transcription 3 (STAT3) and corresponding JAK/STAT signaling [2]. However, the role that aberrant JAK/STAT signaling plays in epigenetic alteration of its downstream targets has yet to be fully elucidated.

Trefoil factors (TFFs), which are secreted by epithelial cells to interact with glycosylated molecules on mucosal surfaces, play an important role in protecting the stomach. Indeed, several studies have shown that TFFs can protect the epithelia against mucosal damage [3,4]. The expression of TFFs stimulates cellular motility as a means of promoting mucosal defense and wound healing [5]. Two out of three TFFs found in the human stomach (TFF1 and TFF2 but not TFF3), have been identified as tumor suppressors [6]. Thus, *TFF1* and *TFF2* (*TFF1/2*) are frequently downregulated in gastric cancer.

GATA6 recognizes the motif (A/T)GATA(A/G) and regulates gene expression, including *TFF1/2*. In humans, GATA1/2/3 are expressed in hematopoietic cells, whereas GATA4/5/6 are expressed in the cardiovascular system and endoderm-derived tissue, including the liver, lung, and pancreas [7]. GATA6 has been shown to regulate a gastric parietal cell-specific gene that encodes H^+^/K^+^-ATPase [8]. Al-azzeh et al. previously found that GATA6 can also activate the expression of TFF1/2 in the stomach [9]. We therefore hypothesized that the downregulation of *GATA6* could lead to the downregulation of *TFF1/2* in gastric cancer.

In this study, we found that the expression level of *GATA6* is inversely correlated with that of STAT3 in gastric cancer patients. A positive correlation was also observed between the expression of *GATA6* and *TFF1/2*. The role of JAK/STAT3 signaling in the suppression of *TFF1/2* through epigenetic silencing of *GATA6* was also explored in this study.

## 2. Results

### 2.1. Inverse Relationship between Expression of STAT3 and GATA6 in Gastric Cancer

**Figure 1:**
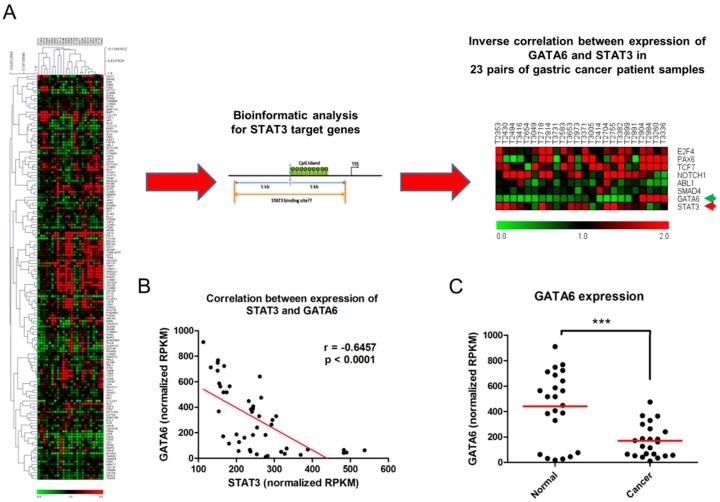
Inverse correlation between the expression of signal transducer and activator of transcription 3 (*STAT3*) and *GATA6* in gastric cancer: (**A**) Heatmap showing the expression profile for 23 pairs of gastric cancer patients investigated in the RNA-Seq experiment (Please refer to Figure S1 for a higher resolution version of the heatmap). Bioinformatic analysis was used to identify potential STAT3 targets by filtering genes with at least one STAT3 binding site within 5 kb of the promoter CpG island. Integration of RNA-Seq and bioinformatic analyses identified several genes, including *GATA6*, which were correlated with the expression of *STAT3*; (**B**) Scatter plot illustrates the inverse correlation between the expression of *STAT3* and *GATA6* among 23 pairs of gastric cancer patients; (**C**) Dot plot shows a significantly higher expression of *GATA6* in adjacent normal tissues than that of cancer tissues (*** *p* < 0.001). Red bar indicates median. TSS: transcription start site; RPKM: reads per kilobase per million.

To identify STAT3 targets that are differentially expressed through the activation of STAT3 in gastric cancer, we performed RNA sequencing (RNA-Seq) by using 23 pairs of gastric cancer patient samples (Figure 1A). We further performed bioinformatic analysis to identify genome-wide CpG island promoters containing STAT3-binding sites and to determine the differentially-expressed genes that are regulated by STAT3 (Figure 1A and Figure S1). Combined results from expression arrays and bioinformatic analyses revealed an inverse correlation between the expression of *STAT3* and *GATA6* in those patient samples (Figure 1B). Interestingly, adjacent normal tissues showed a significantly higher expression of *GATA6* than that of cancer tissues (Figure 1C).

### 2.2. Epigenetic Silencing of GATA6 by Aberrant JAK/STAT Signal Suppresses TFF1/2

**Figure 2:**
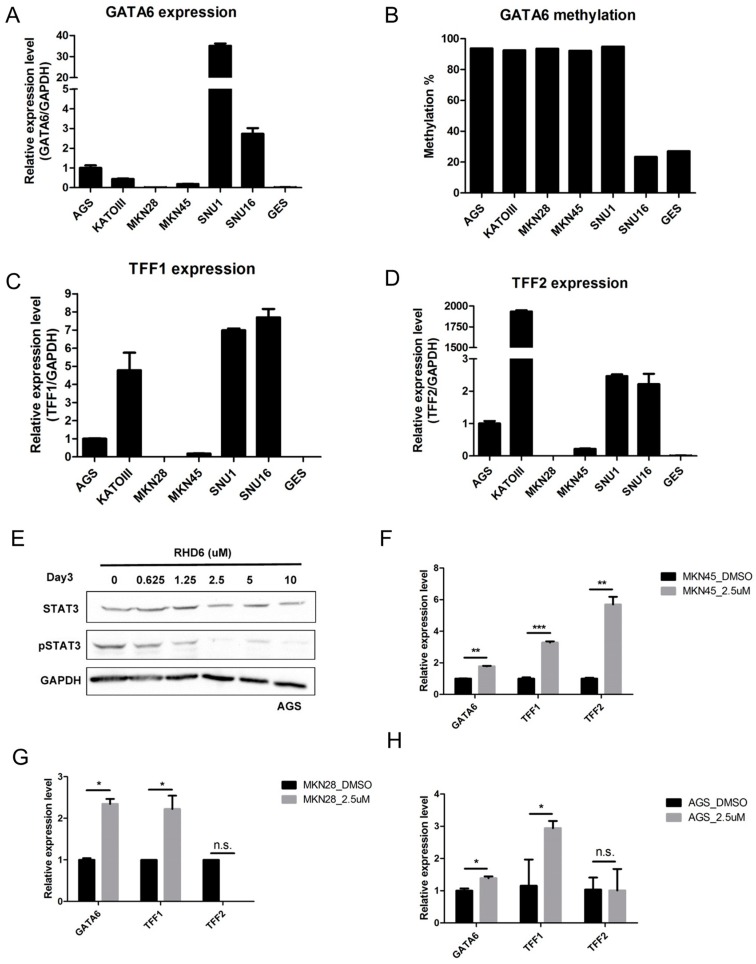
Epigenetic silencing of *GATA6* through the activation of STAT3 suppresses expression of trefoil factors (TFF) 1 and 2 in gastric cancer: (**A**) Relative expression levels of *GATA6* in immortalized gastric epithelial cells (GES) and gastric cancer cell lines as determined by RT-PCR; (**B**) Methylation level of the *GATA6* promoter in gastric cancer cell lines, as determined by bisulfite pyrosequencing. Relative expression levels of (**C**) *TFF1* and (**D**) *TFF2* in the same gastric cancer cell lines, as determined by RT-PCR; (**E**) Western blot analysis showing the protein levels of STAT3 and phosphorylated STAT3 (pSTAT3) in AGS gastric cancer cells treated with various quantities of STAT3 inhibitor rhodium(III) complex 6 (RHD6). The protein level of glyceraldehyde 3-phosphate dehydrogenase (GAPDH) was used as a loading control. Relative expression levels of *GATA6*, *TFF1*, and *TFF2* in (**F**) MKN45, (**G**) MKN28, and (**H**) AGS gastric cancer cells treated with dimethyl sulfoxide (DMSO) or 2.5 μm of RHD6. (*** *p* < 0.001; ** *p* < 0.01; * *p* < 0.05). Each bar represents mean ± standard deviation (SD) of duplicate experiments. n.s.: not significant.

Previous researchers have reported that promoter methylation of *GATA6* was observed in human cancer [10,11]. To determine whether this epigenetic event also occurs in gastric cancer, we examined expression and methylation status in various gastric cancer cell lines. The expression of *GATA6* (Figure 2A,B) was correlated with promoter methylation in all gastric cancer cell lines except for SNU1 and GES cells. Given that GATA6 is a transcription factor of the gastroprotective trefoil genes *TFF1/2* [9], we therefore examined the expression of *TFF1/2* in gastric cancer cell lines. As expected, the expression of *TFF1/2* presented a positive correlation with that of *GATA6* in most of the cells.

The above experiments demonstrate that *GATA6* is epigenetically silenced in gastric cancer. In our previous study on ovarian cancer, we demonstrated that aberrant signaling may lead to the epigenetic silencing of its downstream targets [12,13]. In the current study, gastric cancer cells were then treated with rhodium(III) complex 6 (RHD6) [14], a novel STAT3 inhibitor, to determine whether the suppression of STAT3 would derepress *GATA6* and *TFF1/2*. We found that RHD6 treatment suppressed STAT3 phosphorylation but not total STAT3 in AGS cells (Figure 2E). Importantly, treatment with 2.5 µm of RHD6 restored the expression of *GATA6* and *TFF1/2* in AGS, MKN28, and MKN45 gastric cancer cells (Figure 2F–H). Similar results can be found in AGS cells treated with the JAK inhibitor, AG490 (Figure S2).

### 2.3. STAT3 Binds to the GATA6 Promoter

**Figure 3:**
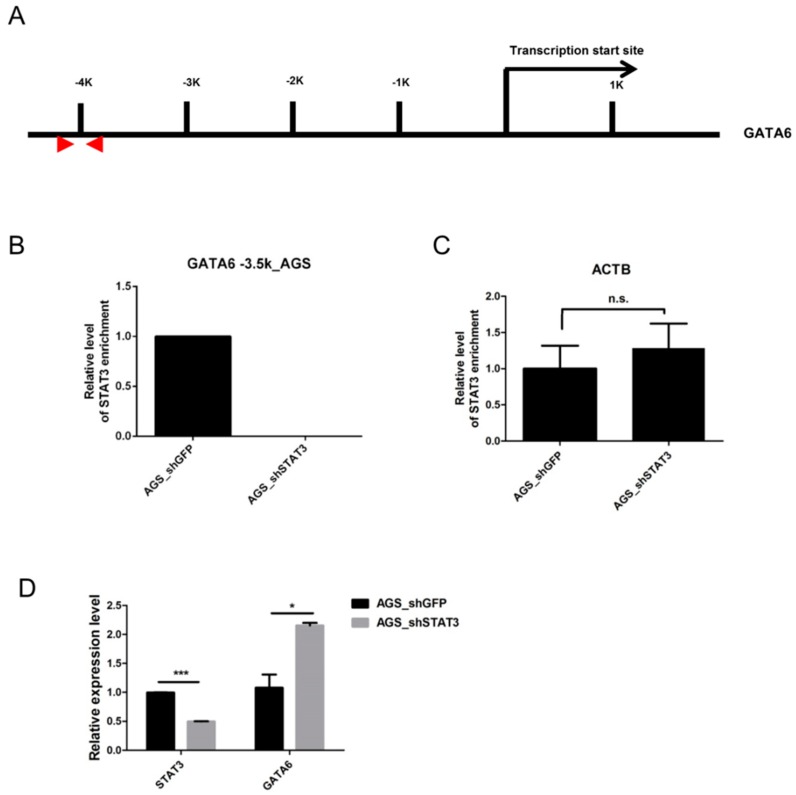
Binding of STAT3 represses *GATA6* expression in AGS gastric cancer cells: Using lentiviral small hairpin RNA (shRNA), chromatin immunoprecipitation (ChIP)-PCR experiments were performed to examine the relative binding of STAT3 to the *GATA6* promoter in AGS cells and cells with depleted STAT3. (**A**) The genomic structure of the *GATA6* promoter and the location of the ChIP-PCR primer (red arrow heads) targeting the putative STAT3 binding site at the upstream promoter region. STAT3 binding was significantly enriched in the (**B**) *GATA6* promoter but not (**C**) β-actin (*ACTB*) promoter (negative control) in AGS cells; (**D**) Depletion of STAT3 significantly restored *GATA6* expression in AGS cells. *** *p* < 0.005, * *p* < 0.05. n.s.: not significant.

To determine whether STAT3 affects the expression of *GATA6* directly, we examined the binding status of STAT3 in AGS cells showing constitutive activation of STAT3 [15,16]. The binding of STAT3 can be observed at the putative STAT3 binding site upstream of the *GATA6* promoter (Figure 3A). The binding at the *GATA6* promoter (Figure 3B) but not β-actin (*ACTB*, negative control, Figure 3C) was disrupted upon STAT3 depletion in AGS cells. Importantly, disruption of STAT3 restored *GATA6* expression in AGS cells (Figure 3D).

### 2.4. Expression of GATA6 Correlates with TFF1/2 in Gastric Cancer Patients

**Figure 4:**
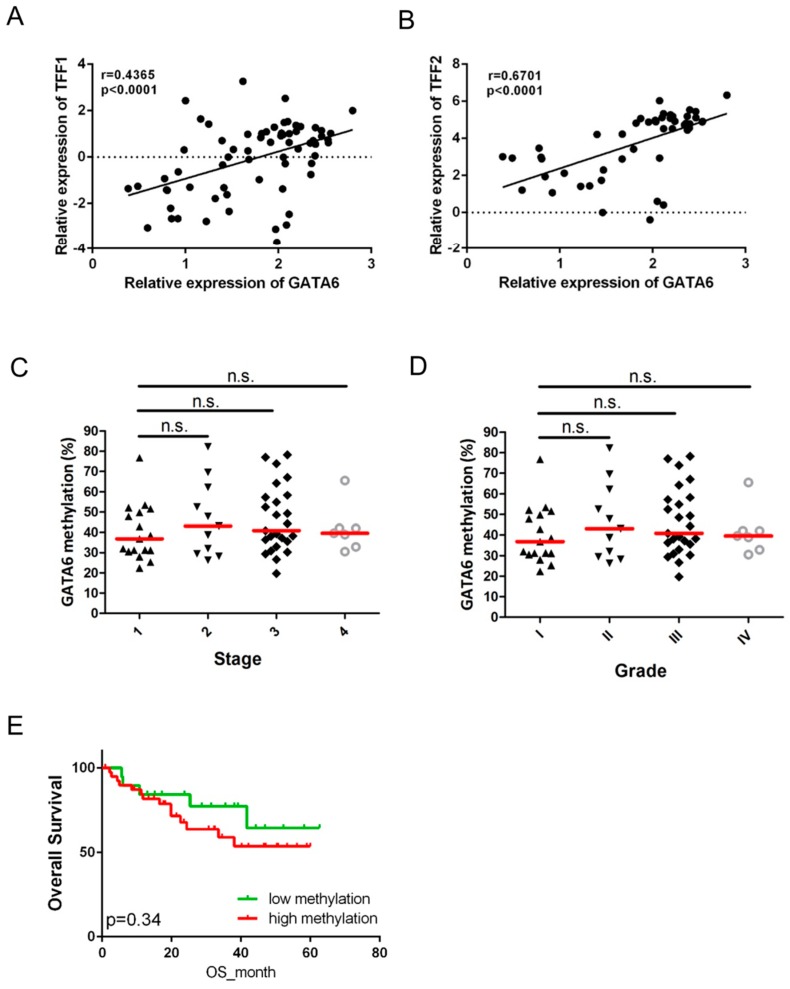
Promoter methylation of *GATA6* is frequently observed in gastric cancer patients. Scatter plots showing the correlation between the expression of *GATA6* and (**A**) *TFF1* and (**B**) *TFF2* among 60 gastric cancer patients. A significant correlation was observed between *GATA6* and *TFF1/2*. The dot plot shows the relationship between methylation level of the *GATA6* promoter and (**C**) tumor stage or (**D**) tumor grade among 60 patients with gastric cancer; (**E**) Results from a Kaplan–Meier analysis which show that gastric cancer patients with higher *GATA6* methylation (red line, >35% methylation, see Materials and Methods) tended to have shorter overall survival than did patients with lower *GATA6* methylation (green line). OS: overall survival. n.s.: not significant.

**Figure 5:**
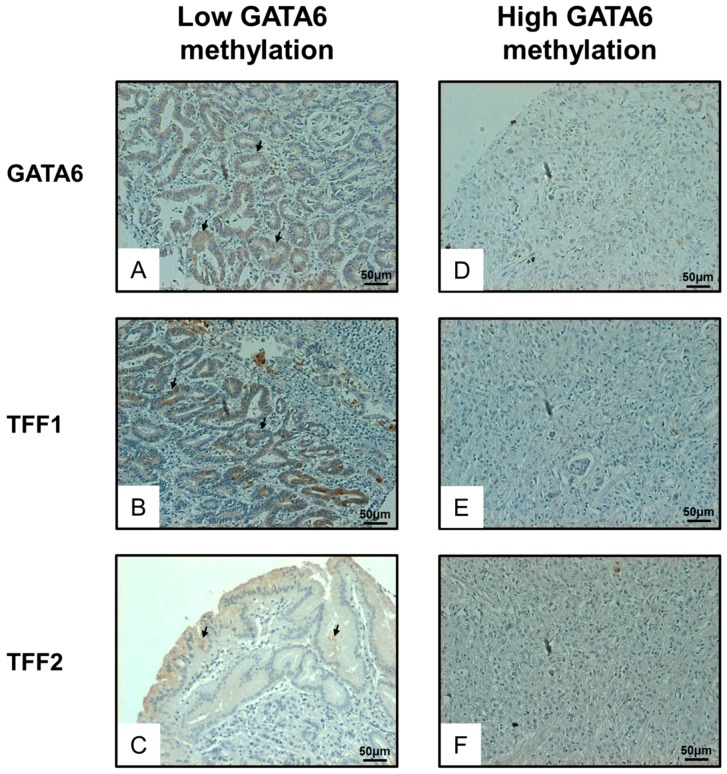
Immunohistochemistry examination of GATA6, TFF1 and TFF2 in gastric cancer patient samples: Samples with low GATA6 methylation (**left** panel) have higher expression of GATA6 (**A**) as well as TFF1 (**B**) and TFF2 (**C**) as indicated by brown color (arrow). In contrast, samples with high GATA6 methylation (**right** panel) have lower expression of GATA6 (**D**), TFF1 (**E**), and TFF2 (**F**). Magnification, 100×.

A significantly positive correlation was observed between the expression of *GATA6* and *TFF1/2* in the full set of patients with gastric cancer (*n* = 60, Table 2, Figure 4A,B). As expected, samples with low *GATA6* methylation but not high *GATA6* expression demonstrated a higher protein expression of GATA6 as well as TFF1/2 (Figure 5). We also examined the clinical significance of *GATA6* methylation (*p* < 0.0001, Figure 4C,D). Despite the fact that *GATA6* methylation was not significantly associated with any clinical parameters (Table 1), patients with higher *GATA6* methylation tended to have shorter overall survival than did those with lower *GATA6* methylation (Figure 4E).

## 3. Discussion

The activation of JAK/STAT signaling plays a crucial role in gastric carcinogenesis [2,17]; however, the role of JAK/STAT signaling in the epigenetic silencing of its targets has not been fully explored. In the current study, we examined the expression profile of STAT3 and its putative targets. Our results demonstrate that there is an inverse relationship between the expression of *STAT3* and *GATA6* in gastric cancer patients. Cell line study also suggests that the expression of *GATA6* may be epigenetically controlled by promoter methylation. Importantly, inhibition of JAK/STAT3 signaling by the novel STAT3 inhibitor derepressed *GATA6* expression in several gastric cancer cell lines suggesting that STAT3 is a transcription repressor for *GATA6*. It is noteworthy that GES cells with a low level of *GATA6* promoter methylation did not express *GATA6*. On the contrary, SNU1 cells showing high levels of *GATA6* methylation also showed a high level of *GATA6* expression (Figure 2A,B). It is thus suggested that other mechanisms such as genetic alteration or histone modifications may be responsible for controlling *GATA6* expression in these cells.

In the trefoil peptide family, TFF1 and TFF2 but not TFF3 are important in maintaining the mucosal integrity of the gastrointestinal tract [4]. Previous studies have demonstrated that TFF1/2 are downregulated in gastric cancer, which suggests that they may be tumor suppressors. In the current study, we observed a positive correlation between the expression of *GATA6* and *TFF1/2* in patients with gastric cancer and in gastric cancer cell lines. Importantly, the inhibition of JAK/STAT signaling by the STAT3 inhibitor restored *GATA6* expression as well as the expression of *TFF1/2* in cancer cell lines.

A previous study demonstrated that GATA6 is an important transcription factor in the transactivation of *TFF1/2* in gastric cancer [9]; however, the current study may provide mechanistic evidence to explain how TFF1 and TFF2 are downregulated by epigenetic silencing of *GATA6* through the activation of JAK/STAT signaling in gastric cancer. It is also noted that the expression of *TFF1/2* did not correlate well with that of *GATA6* in KATOIII cells (Figure 2A–D). The role of factors other than GATA6 in the transcriptional regulation of *TFF1/2* cannot be excluded.

This study also demonstrated that *GATA6* is epigenetically silenced by promoter methylation in patients with gastric cancer. The methylation of the GATA family has previously been described in several types of human cancer [18,19,20]. Specifically, the methylation of *GATA4* and *GATA5* has been frequently observed in gastric cancer, which suggests that these genes may be tumor suppressors [21]. We are therefore the first to report that *GATA6* can be epigenetically silenced by promoter methylation in gastric cancer. Although the methylation of *GATA6* did not correlate with any of the clinical parameters, frequent methylation of *GATA6* was observed in our sample cohort, and patients with higher *GATA6* methylation tended to have shorter overall survival than those with lower *GATA6* methylation. These results suggest that *GATA6* may act as a tumor suppressor in gastric cancer and methylation of *GATA6* may be an early event in gastric carcinogenesis. Methylation of *GATA6* may be able to serve as an early diagnostic marker in gastric cancer. However, further functional and clinical experiments will have to be conducted to test the supposition.

In conclusion, aberrant JAK/STAT signaling may be involved in the epigenetic suppression of *GATA6* expression via promoter hypermethylation. The suppression of *GATA6* may further downregulate *TFF1/2* in gastric cancer. Pharmacological inhibition of STAT3 may restore the expression of *GATA6* and the gastric protective factor TFF1/2. Determining the potential role of the STAT3 inhibitor in the treatment of gastric cancer is worthy of further investigation.

## 4. Materials and Methods

### 4.1. Patient Sampling

Sixty surgical tissue samples of gastric cancer were obtained from the Tissue Bank, Department of Medical Research, Chang Gung Memorial Hospital, Chiayi, Taiwan (Table 2). In this cohort of patients with gastric cancer, the median age at the time of diagnosis was 68.5 years (range: 47–87 years). Out of these samples, 23 tumor-adjacent normal pairs were used to perform RNA-Seq (Table 2). All assessments conducted in this study were approved by the Institutional Review Board (IRB) of the Chang Gung Memorial Hospital, Chiayi, Taiwan (IRB no.: 102-1993B, approval date: 2013/08/08).

### 4.2. Cell Culture

The gastric cancer cell lines AGS, KatoIII, MKM28, MKN45, SNU1, SNU16 and an immortalized gastric epithelial cell line, GES (a kind gift from Dr. Jun Yu, The Chinese University of Hong Kong, Hong Kong), were propagated in Roswell Park Memorial Institute medium (RPMI)-1640 (Invitrogen, Carlsbad, CA, USA) containing 10% fetal bovine serum and incubated at 37 °C under a humidified atmosphere containing 5% CO_2_. Cells were treated with various quantities (control, 0.625 μM, 1.25 μM, 2.5 μM, 5 μM, and 10 μM) of novel STAT3 inhibitor RHD6 [14] for 3 days or 20 μm of AG490 (Sigma, St. Louis, MO, USA) for 24 h. The cells were then harvested for protein and RNA extraction.

### 4.3. DNA Extraction, RNA Extraction, and Quantitative Reverse Transcription-PCR

DNA was extracted using the Tissue & Cell Genomic DNA Purification Kit (Genemark, Taipei, Taiwan). The DNA was eluted in 50 µL distilled water and stored at −20 °C until use. Total RNA from cell lines was extracted using Trizol (Invitrogen) in accordance with the manufacturer’s protocol. Briefly, 1 µg of total RNA was treated with DNase I (amplification grade, Invitrogen) prior to performing first-strand cDNA synthesis using reverse transcriptase (Superscript II RT, Invitrogen). Real-time PCR reactions were conducted using the ABI Step-One real-time PCR system (Applied Biosystems, Foster City, CA, USA) with specific primers (Table 3). The relative expression was calculated using the comparative *C*_t_ method.

### 4.4. Bisulfite Conversion and Pyrosequencing

Bisulfite pyrosequencing was performed as previously described [13]. Briefly, 0.5 µg of genomic DNA was bisulfite-modified using the EZ DNA Methylation Kit (Zymo Research, Orange, CA, USA) in accordance with the manufacturer’s protocol. The bisulfite-modified DNA was subjected to PCR amplification using a tailed reverse primer in combination with a biotin-labeled universal primer. PCR and sequencing primers were designed using PyroMark Assay Design 2.0 software (Qiagen GmbH, Hilden, Germany). *GATA6* transcription start site (−3617 to −3352) was PCR amplified using specific primers (Table 3) in a 25 μL reaction using Invitrogen Platinum™ DNA Polymerase (Invitrogen). Prior to pyrosequencing, 1.5 μL of each PCR reaction was analyzed on 1% agarose gel. Pyrosequencing was performed on the PyroMark Q24 (Qiagen) using Pyro Gold Reagents (Qiagen) in accordance with the manufacturer’s protocol. We measured the methylation level of eleven CpG sites located from −3524 to −3439 bp. The methylation percentage of each cytosine was determined by dividing the fluorescence intensity of cytosines with the sum of the fluorescence intensity for cytosines and thymines at each CpG site. In vitro methylated DNA (Merck Millipore, Billerica, MA, USA) was included as a positive control for pyrosequencing.

### 4.5. RNA-Seq

Total RNA from 23 tumor-normal pairs of gastric cancer tissues (Table 2) was extracted using TRIzol (Invitrogen) in accordance with manufacturer's instructions. RNA-Seq was then performed using Illumina MiSeq (Illumina, San Diego, CA, USA) at the sequencing core of the National Chung Cheng University (Chiayi, Taiwan).

### 4.6. Protein Extraction and Western Blotting

Cells were lysed with 100 μL of PRO-PREP Protein Extraction Solution (iNtRON Biotechnology, Seongnam, Korea) and protein concentrations determined by Bio-Rad Protein Assay kit (Bio-Rad, Hercules, CA, USA), according to the manufacturer’s protocol. Samples and pre-stained protein markers were electrophoresed through 10% sodium dodecyl sulfate-polyacrylamide gel electrophoresis (SDS-PAGE) gels, and then transferred to polyvinylidene fluoride (PVDF) membranes, using the Mini Trans-Blot Electrophoretic Transfer Cell system (Bio-Rad). The membrane was then incubated overnight at 4 °C with primary antibodies, rabbit anti-pSTAT3 (1:1000, Cell Signaling, Beverly, MA, USA), mouse anti-STAT3 (1:1000, Cell Signaling), rabbit anti-GATA6 (1:1000, Abcam, Cambridge, UK), rabbit anti-TFF1 (1:1000, Abcam), mouse anti-TFF2 (1:1000, Abcam) or mouse anti-glyceraldehyde 3-phosphate dehydrogenase (GAPDH) antibodies (1:1000, Thermo Fisher Scientific, Rockford, IL, USA), as diluted in 1× phosphate-buffered saline with Tween (PBST). The membranes were incubated at room temperature for 1 h, with secondary antibodies (Thermo Fisher, anti-mouse, 1:1000 or anti-rabbit 1:1000, diluted in 1× PBST). Proteins were detected by an enhanced chemiluminescence horseradish peroxidase (HRP) substrate detection kit (Merck Millipore) and MiniChemi^TM^ Chemiluminescence imaging system (Sage Creation, Beijing, China).

### 4.7. Immunohistochemical Analysis

Paraffin-embedded gastric cancer tissue sections of selected cases from the above-mentioned patients were dewaxed in xylene and rehydrated in alcohol. Antigen retrieval was performed by heating each section at 100 °C for 25 min in 1× Novocastra^TM^ Epitope Retrieval Solution (pH = 9.0, Leica Biosystems, Newcastle, UK). After 5 min rinses in phosphate-buffered saline (PBS), we followed the protocol of Novolink^TM^ MaxPolymer Detection System (Leica, Wetzlar, Germany) to stain patient samples, with rabbit anti-GATA6 (1:50, Abcam), rabbit anti-TFF1 (1:50, Abcam), or mouse anti-TFF2 (1:50, Abcam) at 4 °C, overnight, followed by secondary antibody containing post primary reagent and HRP-linked polymer. Slides were finally counterstained with hematoxylin.

### 4.8. Statistical Analysis

Overall survival (OS) was assessed using Kaplan–Meier analysis and the log-rank test. Overall survival was defined as the time period between the day of diagnosis and death. A DNA methylation level of 35% was used as the cut-off, because this is the median level of methylation in gastric cancer samples. An unpaired *t*-test was also used to compare parameters in the various groups. All statistical calculations were performed using the SPSS statistical package (version 16.0) for Windows (IBM, Chicago, IL, USA). In this study, a *p* value of <0.05 was considered statistically significant.

## Figures and Tables

**Table 1:** Relationship between clinical parameters and *GATA6* methylation among the 60 patients with gastric cancer.

	GATA6 Methylation % ^1^ (*n*)	*p*
Age		0.163
≥60	50.36 ± 5.48% (10)	
<60	42.82 ± 2.12% (50)	
Sex		0.873
Male	43.87 ± 2.18% (43)	
Female	44.59 ± 4.55% (17)	
Stage		0.497
I–II	42.60 ± 2.99% (28)	
III–IV	45.36 ± 2.72% (32)	
Grade ^2^		0.497
Low	42.60 ± 2.99% (28)	
High	45.36 ± 2.72% (32)	

^1^ Mean methylation % ± standard deviation (SD); ^2^ Pathological grade, low: G1–2; high: G3–4.

**Table 2:** Summary of clinico-pathological data for the gastric cancer patients in this study.

	RNA-Seq (*n* = 23)	Full Set (*n* = 60)
**Age**		
Median	68.5	68.5
Range	47–87	47–87
**Stage**		
I	3	17
II	5	11
III	11	25
IV	4	7
**Grade**		
I	3	17
II	5	11
III	11	25
IV	4	7
**Median survival**	23.53	27.1
***H. pylori***		
positive	16	44
negative	8	16

**Table 3:** Primer sequences used in this study.

	Sequence 5′–3′
**RT Primer**	
STAT3 forward	ACTTTCACTTGGGTGGAGAAGGACAT
STAT3 reverse	CTGCTGCTTTGTGTATGGTTCCA
GAPDH forward	CCCCTTCATTGACCTCAACTACAT
GAPDH reverse	CGCTCCTGGAAGATGGTGA
GATA6 forward	AGCCGGCCCCTCATCAAGCCGCAGAA
GATA6 reverse	AGTTGGCACAGGACAATCCAAGCC
TFF1 forward	CACCATGGAGAACAAGGTGA
TFF1 reverse	TGACACCAGGAAAACCACAA
TFF2 forward	ATGGATGCTGTTTCGACTCC
TFF2 reverse	AGAAGCAGCACTTCCGAGAG
**ChIP-PCR**	
GATA6 forward	CGATCACGGAAAGACACCTT
GATA6 reverse	CCAATGACCGACGAAAGATT
ACTB forward	TGCGTGACATTAAGGAGAAG
ACTB reverse	GCTCGTAGCTCTTCTCCA
**Pyrosequencing**	
GATA6 forward	GGGTGGGGGAGATTTGTAAG
GATA6 reverse	AGCTGGACATCACCTCCCACAACGAAACCTT
	CTCCCTTATACCATATTTCTTCC
GATA6 sequencing	GAGATTTAAATTTAAAGAAAATTAT
UB03 biotin primer	AGCTGGACATCACCTCCCACAACG

RT: reverse transcription; STAT3: signal transducer and activator of transcription 3; GAPDH: glyceraldehyde 3-phosphate dehydrogenase; TFF: trefoil factor; ChIP-PCR: chromatin immunoprecipitation-PCR; ACTB: β-actin.

## Supplementary Materials

Supplementary materials can be found at www.mdpi.com/1422-0067/17/9/1467/s1.
