# Supplementary material for: Aberrant JAK/STAT Signaling Suppresses TFF1 and TFF2 through Epigenetic Silencing of GATA6 in Gastric Cancer
Source: Int J Mol Sci. 2016 Sep 2;17(9):1467. doi: 10.3390/ijms17091467 (PMC5037745; doi:10.3390/ijms17091467)
Supplement: Supplementary file 1 [file ijms-17-01467-s001.pdf]

Supplementary Materials: Aberrant JAK/STAT Signaling Suppresses TFF1 and TFF2 through Epigenetic Silencing of GATA6 in Gastric Cancer

Cheng-Shyong Wu, Kuo-Liang Wei, Jian-Liang Chou, Chung-Kuang Lu, Ching-Chuan Hsieh, Jora M. J. Lin, Yi-Fang Deng, Wan-Ting Hsu, Hui-Min David Wang, Chung-Hang Leung, Dik-Lung Ma, Chin Li and Michael W. Y. Chan

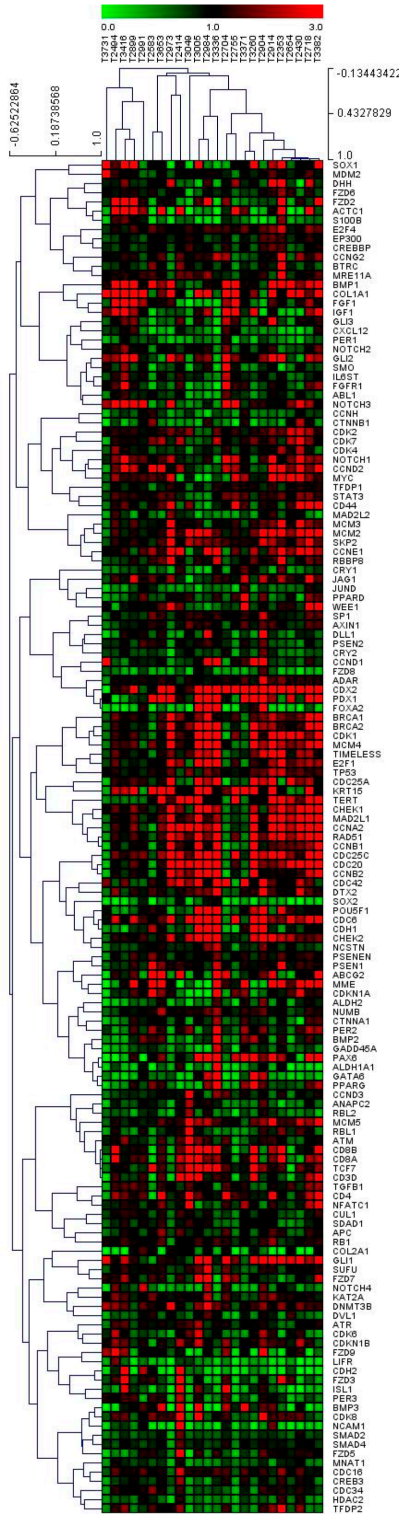

Figure S1. High resolution heatmap image of Figure 1A.

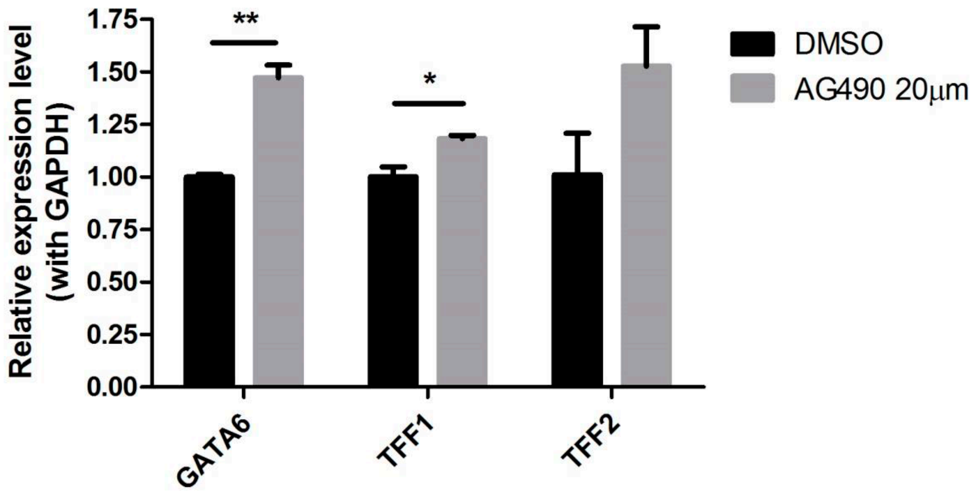

Figure S2. Treatment with AG490 restored the expression of GATA6 and trefoil factors (TFF) 1 and 2 in AGS cells. Cells were treated with 20 μm of AG490 for 24 h and then harvested for RNA isolation and qRT-PCR analysis. ** $p < 0.01$, * $p > 0.05$. GAPDH: glyceraldehyde 3-phosphate dehydrogenase; DMSO: dimethyl sulfoxide.
